# Molecular photoprotection of human keratinocytes *in vitro* by the naturally occurring mycosporine‐like amino acid palythine

**DOI:** 10.1111/bjd.16125

**Published:** 2018-03-25

**Authors:** K.P. Lawrence, R. Gacesa, P.F. Long, A.R. Young

**Affiliations:** ^1^ St John's Institute of Dermatology Faculty of Life Sciences and Medicine King's College London London U.K.; ^2^ Institute of Pharmaceutical Science Faculty of Life Sciences and Medicine King's College London London U.K.

## Abstract

**Background:**

Solar ultraviolet radiation (UVR) induces molecular and genetic changes in the skin, which result in skin cancer, photoageing and photosensitivity disorders. The use of sunscreens is advocated to prevent such photodamage; however, most formulations contain organic and inorganic UVR filters that are nonbiodegradable and can damage fragile marine ecosystems. Mycosporine‐like amino acids (MAAs) are natural UVR‐absorbing compounds that have evolved in marine species for protection against chronic UVR exposure in shallow‐water habitats.

**Objectives:**

To determine if palythine, a photostable model MAA, could offer protection against a range of UVR‐induced damage biomarkers that are important in skin cancer and photoageing.

**Methods:**

HaCaT human keratinocytes were used to assess the photoprotective potential of palythine using a number of end points including cell viability, DNA damage (nonspecific, cyclobutane pyrimidine dimers and oxidatively generated damage), gene expression changes (linked to inflammation, photoageing and oxidative stress) and oxidative stress. The antioxidant mechanism was investigated using chemical quenching and Nrf2 pathway activation assays.

**Results:**

Palythine offered statistically significant protection (*P* < 0·005) against all end points tested even at extremely low concentrations (0·3% w/v). Additionally, palythine was found to be a potent antioxidant, reducing oxidatively generated stress, even when added after exposure.

**Conclusions:**

Palythine is an extremely effective multifunctional photoprotective molecule *in vitro* that has potential to be developed as a natural and biocompatible alternative to currently approved UVR filters.

## Abstract 2

**What's already known about this topic?**

- Mycosporine‐like amino acids (MAAs) are photoprotective molecules found in marine organisms but there are few data on their ability to protect skin cells from the adverse effects of solar ultraviolet radiation (UVR).
- The European Chemicals Agency (ECHA) is concerned about the potential adverse health and ecotoxic effects of eight of 16 commonly used sunscreen filters in Europe. The Environmental Effects Assessment Panel (EEAP) of the United Nations Environment Programme has expressed similar concerns.

**What does this study add?**

- Palythine, an MAA extracted from an edible seaweed, affords photoprotection against a wide range of adverse effects in HaCaT keratinocytes exposed to solar simulating and ultraviolet A radiation. Of note is protection against two types of DNA photolesions; cyclobutane pyrimidine dimers and 8‐oxo‐7,8‐dihydroguanine.
- Palythine is also a potent antioxidant that offers protection even when added after UVR exposure.

**What is the translational message?**

- MAAs should be considered for development as natural biocompatible sunscreens that may address the concerns of the ECHA and EEAP.

**Linked Comment:** https://doi.org/10.1111/bjd.16598.

https://goo.gl/Uqv3dl

Solar ultraviolet radiation (UVR) is a major hazard to many land and shallow‐water based forms of life. Its deleterious effects occur by direct damage to chromophores such as DNA^1^ and other cellular macromolecules, including lipids and proteins, which absorb environmentally relevant UVR (~295–400 nm), or indirectly via generation of reactive oxygen species (ROS).^2^, ^3^, ^4^

The photomolecular events that result in skin cancer, especially keratinocyte cancers, are increasingly understood. Important steps are the generation of DNA photolesions, particularly the cyclobutane pyrimidine dimer (CPD).^5^ This lesion not only generates characteristic UVR signature mutations found in keratinocyte cancers, but is also thought to initiate photoimmunological responses that suppress immunosurveillance of precancerous lesions.^6^, ^7^ UVR‐induced ROS cause oxidatively generated damage to DNA, such as 8‐oxo‐7,8‐dihydroguanine (8‐oxoGua), which is also thought to play a role in skin cancer.^8^ Many photosensitivity disorders are thought to be inflammatory in nature and are triggered by the production of ROS.^9^ Solar UVR also induces gene transcription and protein synthesis that underpin its adverse health effects. Photoageing is caused by UVR‐induction of cutaneous matrix metalloproteinases (MMPs)^10^ that degrade dermal collagens, which are the main structural proteins of the skin.

The incidence of all types of skin cancer continues to increase despite public health campaigns to advise people to reduce solar exposure. Such advice includes shade seeking, avoiding sun when most intense and the use of clothing and sunscreens. The latter contain UVR filters, that is, organic or inorganic compounds that absorb and/or scatter UVR. Typical sunscreen formulations contain several filters with different absorption spectra to cover the solar UVR spectrum. Prospective studies have shown that sunscreen use can inhibit actinic keratoses,^11^, ^12^ keratinocyte cancers^13^ and photoageing^14^ and have some benefit in photosensitivity disorders such as xeroderma pigmentosum.^15^, ^16^

Despite their health benefits, there are emerging ecological concerns with sunscreen use. Most UVR filters are, by design, stable nonbiodegradable molecules. Sunscreen filters in coastal seawaters, can affect phytoplankton and algal growth and cause adverse effects on food trophic levels and the carbon cycle.^17^, ^18^, ^19^ These compounds have also been linked to damage of coral reef ecosystems, promoting viral infections leading to bleaching and coral necrosis.^20^, ^21^, ^22^, ^23^ Many organic filters are lipophilic and so are candidates for bioaccumulation and have been found in the tissues of fish,^24^ dolphins^25^ and birds.^26^ There is evidence that some filters act as endocrine disruptors, displaying oestrogenic and antiandrogenic properties causing changes in secondary sex characteristics in male fish.^27^, ^28^

Certain sunscreen formulations have also been found to cause adverse side‐effects to human health, including contact hypersensitivity,^29^, ^30^ inflammation^31^, ^32^ and systemic accumulation.^24^, ^33^ The Environmental Effects Assessment Panel of the United Nations Environment Programme^34^ recently expressed concern about sunscreen damage to fragile marine ecosystems. In addition, the recently published Community Rolling Action Plan of the European Chemicals Agency included eight of 16 commonly used UVR filters in Europe because of their potential ecotoxicity and adverse impacts on human health. Such concerns are a barrier to sunscreen use, along with a public desire to use more natural and environmentally friendly products.^35^, ^36^

Microorganisms, plants and animals possess complex defence strategies to mitigate UVR‐induced damage. These include DNA repair and antioxidant mechanisms that act through nonenzymatic direct quenching mechanisms, or through the production of enzymatic antioxidants that are synthesized via the cytoprotective Nrf2 pathway.^37^

Many marine organisms synthesize or accumulate water‐soluble mycosporine‐like amino acids (MAAs) that absorb UVR.^38^ MAAs are characterized by either a cyclohexenone or cycloheximine ring conjugated to the nitrogen substituent of an amino acid or amino alcohol. MAAs are thought to afford photoprotection by absorbing UVR energy before it reaches cellular targets, and dissipating this energy as heat. The photoprotective properties of MAAs have been inferred from their high molar extinction coefficients, absorption in the solar UVR region (λ_max_ between 309 and 360 nm) and from circumstantial data.^39^, ^40^ As well as UVR‐absorbing properties, many MAAs have strong antioxidant properties with evidence of both direct chemical quenching and biological Nrf2 activation mechanisms.^41^, ^42^, ^43^

The combined experimental evidence suggests that MAAs have all the necessary characteristics for use as effective biocompatible filters and antioxidants to protect human skin. To date, however, studies on photoprotection have been very limited.^43^ Here we present a study demonstrating that the MAA palythine protects against molecular photodamage in an *in vitro* model of human skin.

## Materials and methods

### Palythine

**Figure 1:**
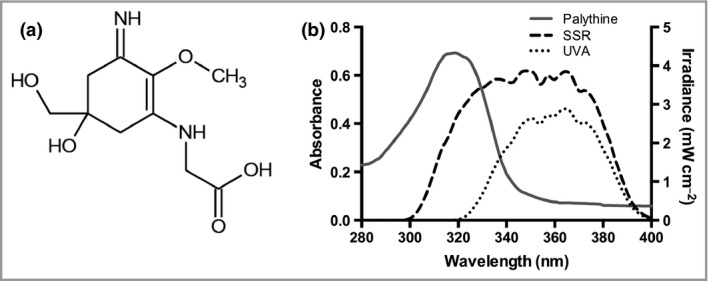
The chemical structure and absorbance spectrum of palythine. (a) The chemical structure of the mycosporine‐like amino acid palythine. (b) The absorbance spectrum of 0·0001% w/v palythine extracted from the red algae *Chondrus yendoi* in phosphate‐buffered saline. There is a strong absorbance in the shorter ultraviolet UVA2 (320–340 nm) and ultraviolet B regions with a peak absorbance of 320 nm. Also displayed are the spectral irradiances of the Solar Light 300W 16S ultraviolet radiation source with the solar‐simulated radiation (SSR) and UVA emission spectra displayed.

Palythine was extracted to analytical grade purity from the red algae *Chrondus yendoi* as described^44^ and diluted to different concentrations (0–10% w/v) in phosphate‐buffered saline (PBS). Purity was confirmed by hydrogen‐ and carbon‐nuclear magnetic resonance. The structure and absorbance spectrum of palythine are displayed in Figure 1.

### Ultraviolet radiation sources and dosimetry

A 300W‐16S xenon arc solar UV simulator (Solar Light, Glenside, PA, U.S.A.) with full solar spectrum UVR and ultraviolet A (UVA) settings, complying with ISO standard 24444 and COLIPA 2006 for solar‐simulated radiation (SSR) and Japanese Cosmetic Industry Association for UVA for the assessment of sunscreen photoprotection was used. The spectral irradiances of the sources were measured using the DM120BC double‐monochromator spectroradiometer (Bentham Instruments, Reading, U.K.) using an integration sphere, calibrated by Public Health England against a U.K. national standard. Irradiance of the sources was routinely measured with a radiometer (IL14000; International Light Technologies, Peabody, MA, U.S.A.) with UVA (SEL033/UVA/TD) and ultraviolet B (UVB) (SEL240/SPS300/W) sensors, which were calibrated against the spectroradiometer.

### Absorbance spectrum

The absorbance spectrum of palythine was measured between 280 and 400 nm using a Spectra Max 384 Plus (Molecular Devices, Sunnyvale, CA, U.S.A.) spectrophotometer.

### Cell culture

The immortalized human keratinocyte cell line HaCaT (ATCC, Manassas, VA, U.S.A.) was cultured to 70–80% confluency in 48‐well plates in Dulbecco's modified Eagle's medium (Invitrogen, Paisley, U.K.), supplemented with 10% (v/v) fetal calf serum, 100 U mL^−1^ penicillin and 100 μg mL^−1^ streptomycin. Cells were incubated in a humidified atmosphere of 5% CO_2_ and 95% air at 37 °C. They were used for all experiments with the exception of the chemical assays [oxygen radical absorbance capacity (ORAC), 2,2‐Diphenyl‐1‐picryl‐hydrazyl (DPPH) and both Nrf2 assays].

### Irradiation procedure

Cells were washed three times in PBS and covered with palythine dissolved in PBS (0–10%). The lid was removed and cells were then irradiated with either SSR or UVA radiation (5–20 J cm^−2^ or 20 J cm^−2^, respectively). After irradiation, palythine solutions were removed and the cells washed a further three times and replaced in media or processed immediately depending on the experimental design.

### Cell viability

Cell viability was measured 24 h after irradiation using the neutral red assay.^45^ Neutral red solution (4 μg mL^−1^ in growth medium) (Sigma‐Aldrich Co., St Louis, MO, U.S.A.) was added to the cells and incubated at 37 °C, 5% CO_2_ for 2 h. Cells were washed three times in PBS to remove excess neutral red solution and then the de‐stain solution (50% v/v ethanol, 49% v/v double distilled water (ddH_2_O), 1% v/v glacial acetic acid) added. Optical density was measured at 540 nm using a Spectra Max 384 Plus spectrophotometer (Molecular Devices). Triton X‐100 (0·1%) was used as a positive control.

### Comet assay to assess DNA photolesions

Cells were treated, gently scraped in PBS and mixed with low melting point agarose (Sigma‐Aldrich) to a final concentration of 0·6% (w/v) and placed in duplicate on microscope slides. The slides were placed into lysis solution (Trevigen, Gaithersburg, MD, U.S.A.) at 4 °C overnight and washed twice in ice‐cold ddH_2_O for 5 min, then in ice‐cold enzyme reaction buffer for 5 min. Slides were incubated with enzyme reaction buffer alone [to assess alkaline labile sites (ALS)], with hOGG1 enzyme (recognizing 8‐oxoGua and 8‐oxo‐7,8‐dihydrodenine, FapyGua and to a much lesser extent, FapyAde lesions) or with T4endoV enzyme (New England Biolabs, Ipswich, MA, U.S.A.) in reaction buffer (to assess CPD and to a lesser extent FapyAde) in a humidified atmosphere at 37 °C for 50 min.^46^ Slides were equilibrated for 40 min in cooled electrophoresis buffer (0·3 mol L^−1^ NaOH, 1 mmol L^−1^ ethylenediaminetetraacetic acid). Electrophoresis was performed for 40 min at 25 V, 300 mA in fresh electrophoresis buffer. Slides were briefly washed in ddH_2_O, dried in 70% (v/v) aqueous ethanol, then stained with propidium iodide solution (2·5 μg mL^−1^) (Sigma‐Aldrich) for 5 min. Finally, slides were washed twice more in ddH_2_O and left to dry in the dark overnight.^47^ H_2_O_2_ treatment (0·03%) (Sigma‐Aldrich) for 5 min was used as the positive control to fragment DNA.

Comets were photographed with a Zeiss Axiophot fluorescent microscope at ×20 magnification (Zeiss, Thornwood, NY, U.S.A.) and percentage DNA present in the tail was measured for at least 50 comets per condition using Comet Score Pro software (Tritek Corp, Sumerduck, VA, U.S.A.).

### Immunocytochemistry – immunofluorescence for cyclobutane pyrimidine dimer

Cells were washed, then fixed in 2% (v/v) paraformaldehyde with 0·5% (v/v) Triton X‐100 in PBS for 30 min at 4 °C. DNA was then denatured by incubation in 2 mol L^−1^ HCl for 10 min at 37 °C. Nonspecific sites were blocked using blocking buffer [20% (v/v) goat serum and 0·1% (v/v) Triton X‐100 in PBS] for 30 min at room temperature. Anti‐CPD antibody (Clone TDM‐2) (Cosmobio, Tokyo, Japan) was added at 1 : 1000 in blocking buffer for 1 h at room temperature. Alexafluor 488 (Thermo Fisher Scientific, Waltham, MA, USA) was diluted in blocking buffer (10 μg mL^−1^) and incubated for 1 h at room temperature and finally 4′,6‐diamidino‐2‐phenylindole (DAPI) (Thermo Fisher Scientific) was added for 10 min. Washing was carried out (3 × 5 min) between each step. Image capture of cells was carried out using a Ziess Axio‐Observer Z1 Microscope (Carl Zeiss, Cambridge, U.K.) with AxioVision V.4.8 software (Carl Zeiss).

Image analysis was carried out using Cell Profiler v.2.1.1 (Broad Institute, Cambridge, MA, U.S.A.), gating around the nucleus of each cell and the relative mean green intensity (CPD staining) of each nucleus was measured (~150 nuclei measured per condition). The mean of the nine pictures was determined and used as the end point.

### Detection of oxidizing species

Carboxy‐2ʹ,7ʹ‐dichlorodihydrofluorescein diacetate (carboxy‐H_2_DCFDA) was used to assess ROS and other oxidizing species (including reactive nitrogen species, free radicals, nitric oxide and peroxides).^48^ HaCaT cells were irradiated as above, or incubated with palythine after irradiation. Cells were then incubated with 10 μmol L^−1^ carboxy‐H_2_DCFDA (Invitrogen) in PBS for 30 min in the dark at 37 °C, 5% CO_2_. Cells were washed in PBS, trypsinized for 10 min at 37 °C, centrifuged at 1200 ***g*** for 5 min at room temperature and resuspended in PBS and counterstained with DAPI for analysis by fluorescence‐activated cell sorting using a Becton Dickinson FACSAria II (Becton Dickinson, Franklin Lakes, NJ, U.S.A.). Cells were gated to only analyse live cells (DAPI negative) and the average mean green intensity per condition was then plotted from at least 10 000 measured events. Analysis was carried out using FlowJo 8.7 (FlowJo, Ashland, OR, U.S.A.). Menadione (50 μmol L^−1^) (Sigma‐Aldrich) was used as a positive control.

### 2,2‐Diphenyl‐1‐picryl‐hydrazyl radical scavenging assay

A 100 μmol L^−1^ stock of DPPH was prepared in methanol and 187·5 μL was added to the wells of a 96‐well plate. Serial dilutions of test compounds were prepared in ddH_2_O, and 12·5 μL was added to each well and mixed. The plate was protected from light and placed on a shaker at room temperature for 30 min. Absorbance was measured at 517 nm using a Spectra Max 384 Plus spectrophotometer (Molecular Devices). Each condition was tested in triplicate.

### Oxygen radical absorbance capacity antioxidant assay

The ORAC assay uses the thermal decomposition of the chemical 2,2′‐azobis‐(2‐amidinopropane dihydrochloride) to generate carbon‐centred free radicals. These are then able to react with oxygen to produce alkoxyl and peroxyl radicals.^49^ These radicals subsequently oxidize fluorescein, resulting in a decrease in the fluorescence signal which is measured over time.^50^ The ORAC assay was carried out with the ORAC Antioxidant Assay Kit (Zenbio, Raleigh, NC, U.S.A.) according to the manufacturer's instructions as described in Data S1 (see Supporting Information).

### Fluorescence polarization assay

The fluorescence polarization (FP) assay tests the ability of compounds to compete with a fluorescein‐labelled peptide (fluorescein‐[β‐ala]DEETGEF‐OH) designed to mimic Nrf2 and to bind to the Kelch‐repeat domain of Keap1 protein. The FP assay was carried out as previously described^51^ and described in detail in Data S1 (see Supporting Information).

### Thermal shift assay

The thermal shift assay measures the change in denaturation temperature between the free Keap1 and ligand‐bound Keap1 protein. The assay was carried out as previously described^52^ and described in detail in Data S1 (see Supporting Information).

### RNA extraction and quantitative reverse transcription polymerase chain reaction

RNA extraction was carried out 12 h post SSR exposure using the mirVana miRNA Isolation kit (Life Technologies, Paisley, U.K.) according to the manufacturer's protocol. RNA was reversely transcribed to cDNA using the High Capacity cDNA Reverse Transcription Kit (Applied Biosystems, Paisley, U.K.). Quantitative polymerase chain reaction was performed using TaqMan Gene Expression Assays (Applied Biosystems, Foster City, CA, U.S.A.) according to the manufacturer's protocols. *GAPDH* was used as the housekeeping gene. Gene fold change was measured using the ΔΔCt method.^53^ Gene selection was based on *in vivo* human studies.^54^

### Statistical analysis

All data are expressed as the mean ± SD where *n* ≥ 3 experimental replicates. Statistical analyses were carried out using Graphpad Prism 6.0 (Graphpad Software, San Diego, CA, U.S.A.) or Origin Pro software (Origin Lab, Northampton, MA, U.S.A.) and were evaluated using Student's *t*‐test, anova, linear and nonlinear regression. A *P*‐value of < 0·05 was considered significant.

## Results

### Palythine absorbs in the ultraviolet radiation region

The absorbance spectrum of palythine (Fig. 1b) demonstrates significant absorbance mainly in the UVB region (λ_max_ = 320 nm), with almost no absorption beyond 340 nm.

### Palythine inhibits solar‐simulated radiation‐induced cell death

**Figure 2:**
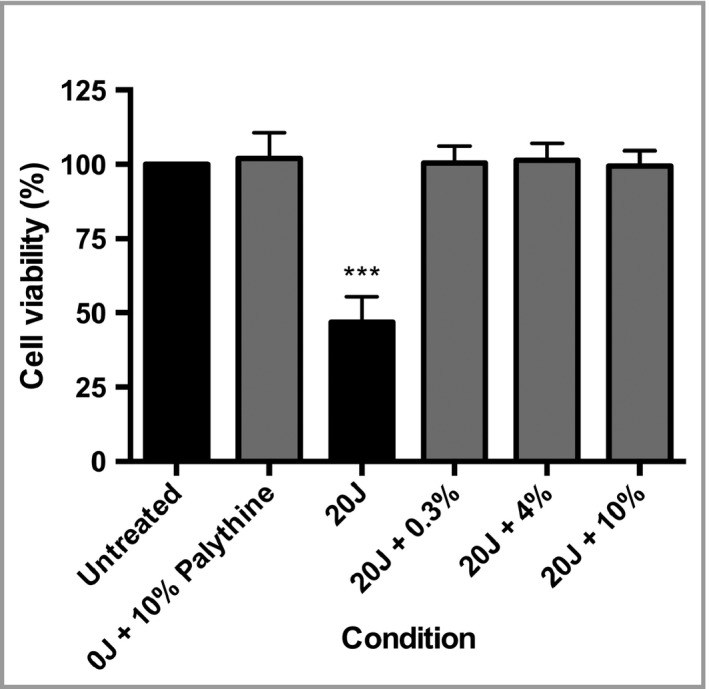
HaCaT keratinocytes were significantly protected by palythine from solar‐simulated radiation (SSR)‐induced cell death. HaCaT keratinocytes were untreated, exposed to 20 J cm^−2^ of SSR with phosphate‐buffered saline (PBS) alone, 0·3% (w/v), 4% (w/v) or 10% (w/v) of palythine or 10% (w/v) palythine without exposure to ultraviolet radiation. Cell viability was measured 24 h later by the neutral red assay. Columns represent the mean + SD (*n* = 3). Palythine prevented SSR‐induced cell viability reduction. ***P < 0·001.

Palythine (0·3–10% w/v) inhibited SSR (20 J cm^−2^) induced cell death (Fig. 2). There was no significant difference between unexposed cells and those treated with palythine (10% w/v), indicating a lack of toxicity.

### Palythine protects against ultraviolet radiation‐induced DNA damage

**Figure 3:**
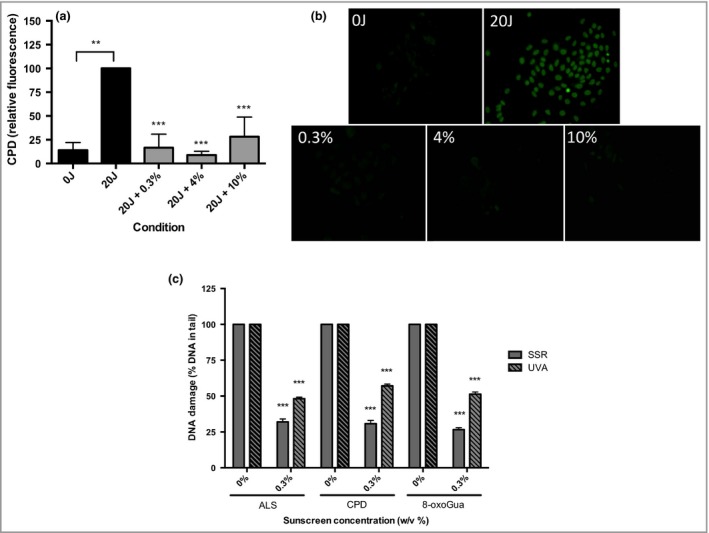
HaCaT keratinocytes were significantly protected from solar‐simulated radiation (SSR) and ultraviolet A (UVA)‐induced cyclobutane pyrimidine dimer (CPD), 8‐oxo‐7,8‐dihydroguanine (8‐oxoGua) and alkali labile sites (ALS) by palythine at a range of concentrations. (a) HaCaT keratinocytes were untreated, exposed to ultraviolet radiation (UVR) (20 J cm^−2^ SSR) with 0·3%, 4% or 10% w/v palythine. CPDs were measured immediately after exposure using immunocytochemistry immunofluorescence. Columns represent mean + SD (*n* = 3). Cells irradiated without palythine showed a significant increase in CPD production (*P* = 0·003, paired *t*‐test) compared with unirradiated control. Palythine at 0·3–10% w/v showed a significant reduction in CPD compared with irradiated control (*P* < 0·001, one‐way anova with Dunnett's multiple comparisons test). There was no significant difference in protection between any concentration of palythine (*P* = 0·332). (b) Typical fluorescent images for each condition. (c) Cells were irradiated with 5 J cm^−2^ SSR or 20 J cm^−2^ UVA radiation with or without 0·3% of palythine and ALS, CPD and 8‐oxoGua production were measured for both spectra tested. The irradiated control was set at 100% for a given experimental run. The effect of palythine is given relative to the control. For SSR – ALS: P = 0·0006, *n* = 3; CPD:* P* < 0·001, *n* = 3; 8‐oxoGua: *P* < 0·001, *n* = 3. For UVA – ALS:* P* < 0·001, *n* = 3; CPD:* P* < 0·001, *n* = 3; 8oxoGua *P* < 0·001, *n* = 3, paired *t*‐test. **P < 0·01; ***P < 0·001.

SSR significantly increased CPD (*P* = 0·003) (Fig. 3a, b) compared with the unirradiated control. Palythine, at all concentrations (0·3–10%), significantly protected against CPD (*P* < 0·001). This was confirmed with the comet assay (Fig. 3c), which showed a significant reduction in ALS, CPD and 8‐oxoGua after exposure to 5 J cm^−2^ with 0·3% palythine compared with PBS alone (*P* ≤ 0·006). There was also significant protection by palythine (0·3%) against the same DNA lesions after exposure to 20 J cm^−2^ of UVA radiation (*P* < 0·001). These data show that palythine protects against different types of DNA photodamage.

### Palythine inhibits solar‐simulated radiation‐induced gene expression

**Figure 4:**
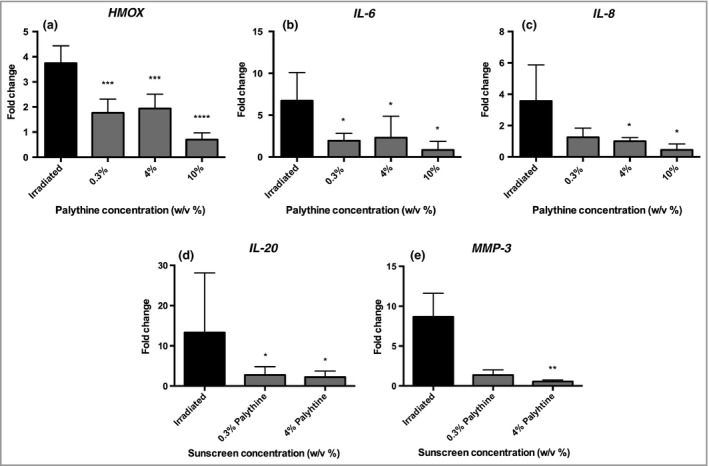
Palythine provided significant protection from solar‐simulated radiation (SSR)‐induced gene expression changes in HaCaT keratinocytes. HaCaT keratinocytes were untreated, exposed to 5 J cm^−2^ of SSR with phosphate‐buffered saline (PBS) alone, 0·3%, 4% and in some cases 10% of palythine. Gene expression was measured 12 h after exposure by quantitative polymerase chain reaction (qPCR) assessing the following genes: *HMOX1*,*IL*‐*6*,*IL‐* *8*,*IL‐* *20* and *MMP‐* *3*. Columns represent the mean + SD (*n* = 5 experimental replicates). Palythine provided significant protection compared with irradiated cells for all genes tested at all concentrations (*P* < 0·05, one‐way anova), with the exceptions of 0·3% palythine against *IL*‐*8* and *MMP‐3*. *P < 0·05; **P < 0·01; ***P < 0·001; ****P < 0·0001

Palythine (0·3–10% w/v) inhibited SSR‐induced (5 J cm^−2^) expression of genes encoding inflammatory cytokines [interleukin (IL)‐8, IL‐6, IL‐20], the oxidative stress response enzyme heme oxygenase 1 (HMOX1), and the matrix remodelling enzyme marker of photoageing (MMP‐3) (Fig. 4). This inhibition was significant (*P* < 0·05) with the exception of IL‐8 and MMP‐3 with palythine at 0·3% w/v. The data show that palythine protects against SSR‐induced markers of inflammation, oxidatively generated stress and photoageing.

### Palythine is an antioxidant

**Figure 5:**
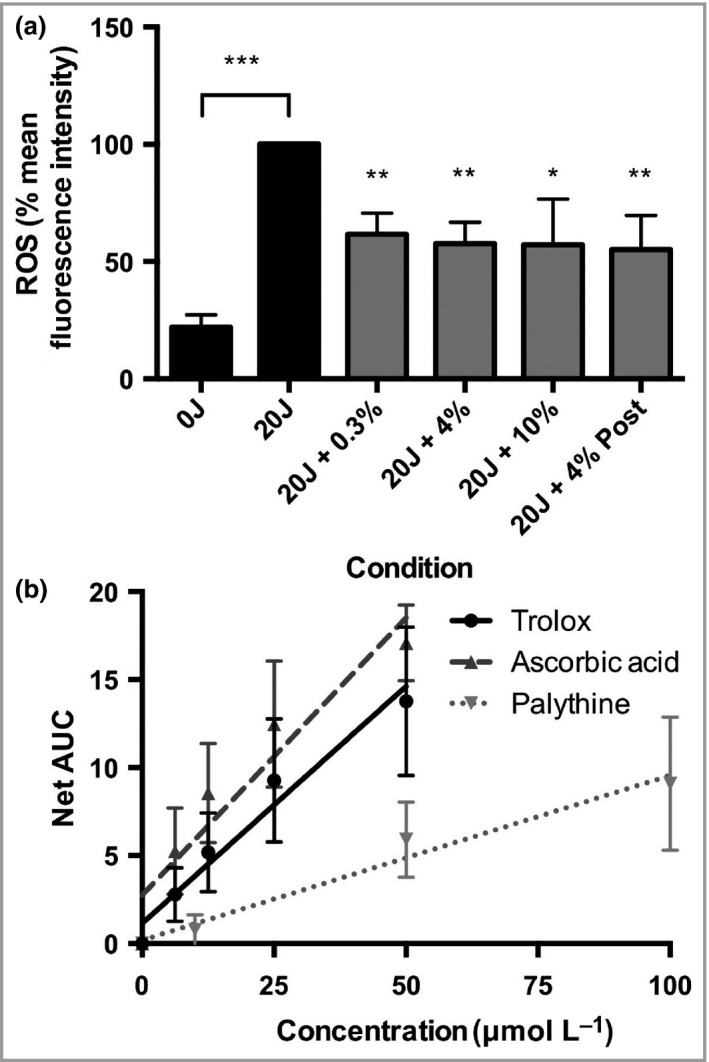
Palythine provided significant protection from ultraviolet radiation‐induced reactive oxygen species (ROS) production and exhibited antioxidant properties *in vitro* through chemical quenching. (a) HaCaT keratinocytes were untreated, exposed to 20 J cm^−2^ of solar‐simulated radiation (SSR) with phosphate‐buffered saline (PBS) alone, 0·3%, 4% or 10% palythine or 4% palythine after exposure. The levels of oxidizing species were measured by fluorescence‐activated cell sorting to record the mean fluorescent intensity for each condition after cells were treated with carboxy‐2ʹ,7ʹ‐dichlorodihydrofluorescein diaceta, and the percentage change compared with the irradiated control was plotted. Columns represent the mean + SD (*n* = 5 experimental replicates). Palythine provided significant protection compared with irradiated control at all concentrations (*P* < 0·05, one‐way anova). (b) Palythine solutions (0–100 μmol L^−1^) were analysed for their ability to quench the ROO˙ radical as a measure of antioxidant capability activity using the oxygen radical absorbance capacity assay and compared with known antioxidants Trolox (0–50 μmol L^−1^) and ascorbic acid (0–50 μmol L^−1^). Fluorescence degradation over 30 min was assessed for each concentration and the area under the curve (AUC) calculated and plotted. Linear regression was carried out and significance of the slope was calculated (Trolox: *y* = 0·9695*x* + 1·154, *P* = 0·0008; ascorbic acid: *y* = 0·3154*x* + 2·760, *P* < 0·001; palythine: *y* = 0·09372 + 0·1932, *P* = 0·004; linear regression analysis, *n* = 3). Slopes were compared to calculate the relative activity of palythine compared with controls (palythine activity = 34·8% of ascorbic acid and 29·1% of Trolox); **P < 0·01; ***P < 0·001.

SSR‐irradiated cells (20 J cm^−2^) had significantly more oxidizing species, than the unirradiated control (Fig. 5a). Palythine (0·3–10% w/v) added prior to irradiation significantly inhibited the production of oxidizing species *(P* < 0·05). A similar result was observed when palythine (4% w/v) was added immediately after irradiation (*P* < 0·05).

The chemical antioxidant mechanisms of palythine were investigated using the DPPH and ORAC assays (Fig. 5b). Palythine was compared with ascorbic acid [half maximal effective concentration (EC_50_) = 21·3 ± 3·0 μmol L^−1^] and α‐tocopherol (EC_50_ = 11·1 ± 3·4 μmol L^−1^) as established positive controls in the DPPH assay. Palythine demonstrated some activity (EC_50_ = 714·3 ± 73·9 μmol L^−1^) but this was much lower than controls. The ORAC positive controls were with Trolox (a water‐soluble analogue of α‐tocopherol) and ascorbic acid. Palythine showed significant activity (*P* = 0·004), comparable with the controls (34·8% and 29·7% of Trolox and ascorbic acid, respectively).

Palythine was also tested for activation of Nrf2‐mediated cytoprotection by *in vitro* FP and thermal shift assays for competitive inhibition of Keap1‐Nrf2 binding. Palythine demonstrated no antagonistic effect, even at the highest concentration of 100 μmol L^−1^ in the FP assay. Palythine (100 μmol L^−1^) bound poorly to the Kelch‐repeat domain of Keap1 protein in the thermal shift assay, as indicated by its low ΔTm = 0·09 ± 0·05 °C, while the positive control [β‐Ala]‐DEETGEF‐OH peptide had a high ΔTm of 3·91 ± 0·04 °C at 50 μmol L^−1^.

## Discussion

Sunscreen use is widely advocated for the prevention of the acute and long‐term effects of solar UVR. Global regulatory bodies approve the formulations' component synthetic organic molecules or inorganic and organic pigments. There is increasing concern (discussed above) that some of these agents are ecotoxic. This has initiated the exploration of natural biocompatible sunscreens, which have evolved under conditions of extreme insolation.

This study determined whether palythine, a natural marine MAA, satisfies the necessary criteria to provide a feasible alternative to synthetic sunscreens. Excellent photostability is a critical requirement of UVR filters, but poor photostability has been a concern with some commercially available synthetic filters.^55^ MAA photostability has been studied extensively, and palythine has been found to be extremely photostable in air saturated aqueous solutions^56^ and in distilled and sea water, even in the presence of powerful photosensitizers.^57^ We previously demonstrated that palythine is very photostable and retains over 95% of its UVR‐absorbance properties after irradiation of up to 40 standard erythema doses of SSR, which is equivalent to around a full day of U.K. summer sun.^43^

The absorption of UVR by skin chromophores initiates the formation of mutagenic DNA‐photoproducts that cause the acute and long‐term damage leading to photoageing and carcinogenesis. This study investigated clinically relevant molecular biomarkers associated with solar UVR‐induced damage to human skin.^10^, ^58^ The studies reported were done with environmentally and physiologically relevant UVR exposure. For example, 20 J cm^−2^ SSR is equivalent to about 1·5 h of peak U.K. summer sun,^59^ equivalent to about four minimal erythema doses in fair‐skinned individuals.^60^

HaCaT keratinocytes were selected as the model for numerous reasons. Firstly, most MAA studies have been carried out on fibroblasts. These are less relevant for skin photoprotection as they are not relevant in photocarcinogenesis. Furthermore, there is a history of using HaCaTs for photobiology and photoprotection studies^61^, ^62^ and good correlation has been demonstrated between HaCaTs and primary keratinocytes and *in vivo* models^63^, ^64^ (including unpublished data from our laboratory) including gene expression. However, there may be some differences between HaCaTs and normal human keratinocytes or whole skin.

Figure 2 shows that SSR reduced cell viability to ~50% of unexposed control cells, and that palythine significantly protects against cell death across a range of concentrations. Complete protection at concentrations as low as 0·3% is advantageous because most sunscreens contain a combination of organic and/or inorganic UVR filters at concentrations between 1% and 25%. Palythine was also shown to have no effect on cell viability when tested at 10% for 24 h.

CPD are readily induced by SSR as shown immunocytochemically in Figure 3a, b. The addition of palythine, at all concentrations (0·3–10% w/v) resulted in a highly significant reduction of CPD (with two independent assays), comparable with the unirradiated control. The comet assay (Fig. 3c) also confirmed that palythine, at all concentrations, significantly reduced SSR‐induced ALS and 8‐oxoGua. Comparable results were seen with UVA, which is the major component (~95%) of solar UVR. Protection by palythine against UVA‐induced DNA damage is somewhat unexpected because of its low *in vitro* UVA protection factor (UVAPF) (Table S1; see Supporting Information). This suggests that the palythine's antioxidant properties play a major role in the prevention of UVA‐induced DNA damage. These results are the first demonstration of MAA protection against direct and oxidatively induced DNA damage by UVA and SSR. They are also in accordance with a study by Torres *et al*.,^65^ who reported that the MAA collemin A protects against UVB‐induced CPD in HaCaT keratinocytes.

The selection of genes was based on human *in vivo* data from our laboratory.^54^ Palythine inhibited the expression of genes associated with antioxidant activity, cytokines associated with inflammation/immunoregulation and photoageing (Fig. 4), with the exception of *IL‐8* and MMP‐3 (at 0·3% w/v). In general, there was no advantage with palythine at higher concentrations. The *MMP‐3* data support a role for protecting against photoageing that has also been reported for MAA (porphyra‐334) against MMP‐1 after UVA exposure of skin fibroblasts.^66^

UVR‐induced ROS in skin have been well‐documented.^2^, ^3^ Most evidence for the antioxidant properties of MAAs has come from nonbiological, chemical assays.^67^, ^68^ Figure 5a shows that palythine significantly reduces SSR‐induced oxidizing species in a biological system. Studies in human keratinocytes *in vitro* have demonstrated that ROS are generated nonphotochemically for 15 min after UVR exposure.^4^ In this study, palythine was also added immediately after UVR exposure in order to distinguish between its UVR filtering and antioxidant properties. The results show that palythine is equally effective under both conditions, confirming its antioxidant properties although not excluding its benefit as a filter. These results support a recent study that showed preincubation for 24 h with an MAA (porphyra‐334) significantly reduced UVA‐induced ROS in human skin fibroblast CCD‐986sk cells.^66^

Free radical quenching and antioxidant mechanisms were investigated in four ways. The DPPH assay is a measure of free radical quenching and the ORAC assay measures antioxidant capability; however, the latter is nonspecific and free radicals are also produced in the process of generating the pro‐oxidants. The ORAC assay demonstrated that palythine is an effective antioxidant, taken from the fact there was low activity in the DPPH assay, suggesting that antioxidant activity was the more important mechanism. The antioxidant capacity was around 30% of established powerful antioxidants. Although less potent, palythine is much more photostable than ascorbic acid (e.g. ascorbic acid degrades 70× faster with exposure to UVR compared with an unirradiated control)^69^ and provides only minimal absorption in the solar UVR region.^70^ These data suggest that palythine is a much more effective antioxidant, even under conditions of high insolation.

It has previously been demonstrated that the MAAs porphyra‐334 and shinorine inhibit Keap1‐Nrf2 binding *in silico* 41 and that porphyra‐334 activates Nrf2‐regulated genes in human skin fibroblast cell culture.^42^ This was not the case for palythine using both the FP and thermal shift assays. This strongly suggests that palythine's antioxidant properties are chemical rather than biological, and that different MAAs have distinct antioxidant mechanisms.

A high extinction coefficient [ε^(m)^] is an essential requirement of a UVR filter that enables its use at a low concentration, reducing costs and potential adverse side‐effects. The ε^(m)^ is the basis for the *in vitro* SPF and UVA‐PF test methods.^71^ Palythine photoprotection is likely to be in part because of its high ε^(m)^ (36 947·7 ± 2 238·6, Table S1; see Supporting Information), which confirms another report.^72^ This value is in the range of commonly used synthetic filters which vary from ε^(m)^ = 4900 for octyl salicylate to ε^(m)^ = 120 000 for ethylhexyl triazone.^73^

The *in vitro* SPF and UVAPF were calculated (Table S1 and Data S1; see Supporting Information), demonstrating relatively high SPFs for a single molecule that was dependent on palythine concentration. Most sunscreens contain mixtures of several filters, each in the range of 1–25%; however, palythine for example at 10% has an SPF of 17·9, despite its minimal UVA absorption (UVAPF = 1·6). This shows that using palythine alone is not enough to pass the stringent sunscreen requirements for UVA protection. The selection of palythine concentrations for the experiments was based on those used for currently approved UVR filters in sunscreens. Unexpectedly, no palythine dose–response relationships were observed for most of the biological assays. This may be a consequence of its potent antioxidant properties that synthetic filters do not possess. Such properties would have no influence on the *in vitro* SPF calculations and may compensate for spectral shortcomings in the UVA region.

In conclusion, the data show that palythine, even at low concentrations, significantly reduces the most clinically relevant forms of solar UVR‐induced damage in an *in vitro* skin model. Unlike currently approved UVR filters, palythine combines photostability, UVR filtering and antioxidant properties in a single molecule. There are challenges in producing and developing MAAs as sunscreens that have recently been reviewed.^43^ However, this suggests that MAAs have the potential to be developed as effective biocompatible UVR filters that may appeal to the public as natural products.^35^ This would require studies to assess the ability of palythine and other MAAs to inhibit erythema and molecular damage *in vivo*. The data also suggest that MAAs may have a role in after‐sun preparations.

## Supporting information


**Data S1.** Supplementary methods for the oxygen radical absorbance capacity fluorescence polarization and thermal shift assays and the molar extinction coefficient and *in vitro* sun protection factor calculations.

**Table S1.** The molar extinction coefficient and *in vitro* sun protection factor of palythine.
