# Supplementary material for: Molecular photoprotection of human keratinocytes in vitro by the naturally occurring mycosporine‐like amino acid palythine
Source: Br J Dermatol. 2018 Mar 25;178(6):1353–63. doi: 10.1111/bjd.16125 (PMC6032870; doi:10.1111/bjd.16125)
Supplement: Supplementary file 1 — Data S1. Supplementary methods for the oxygen radical absorbance capacity fluorescence polarization and thermal shift assays and the molar extinction coefficient and in vitro sun protection factor calculations. [file BJD-178-1353-s001.docx]

**File S1** Supplementary methods for the oxygen radical absorbance capacity fluorescence polarization and thermal shift assays and the molar extinction coefficient and in vitro sun protection factor calculations.

***ORAC assay method***

The ORAC assay was carried out with the ORAC Antioxidant Assay Kit (Zenbio, North Carolina, USA) according to manufacturer’s instructions. Trolox standards were prepared in the assay buffer (100-0μM) along with serial dilutions of the test compounds. 150μl of the fluorescein working solution was added to the inner wells of a 96 well plate, with 25μl of each of the standards or test compound in duplicate, and the plate incubated at 37^o^C for at least 15 minutes. The 2,2’-azobis-2-methyl-propanimidamine dihydrochloride (APPH) working solution was added to each well (25μl) to start the reaction. Fluorescence was measured in a preheated incubation chamber (37^o^C) using a Spectra Max 384 Plus spectrophotometer (Molecular Devices; Sunnyvale, California, USA) with excitation/emission = 485/530nm immediately and then every minute for 30 minutes. Standard curves were generated for each compound and the area under the curve (AUC) calculated. Each compound tested was expressed as a Trolox equivalent concentration.

***Fluorescence polarization assay method***

A solution containing 1 nM of the fluorescent peptide FITC-β-DEETGEF-OH and 200nM of the Keap 1 Kelch domain in PBS (pH 7.4) was prepared and plated into a 96 well plate. Test solutions (0-100µM) were added to the wells up to a final volume of 100µL and incubated in the dark at room temperature for 1hr. FP was measured using a Perkin Elmer EnVision™ Multilabel Plate Reader (Perkin Elmer, Beaconsfield, UK). Each condition was tested in triplicate and fitted to a standard dose-response curve by non-linear IC50 values were determined.

***Thermal shift assay***

A detection dye SYPRO® orange (5X) and 5µM of Keap1 Kelch domain protein was prepared in PBS (pH 7.4) and plated into a 96 well plate. Test solutions (0-100µM) were added to the wells up to a final volume of 40µL and incubated in the dark at room temperature for 1hr. The plate was then placed into a 7500 Real Time PCR machine (Applied Biosystems by Life Technologies, Carlsbad, USA) and heated uing a standard protein melting protocol ^1^. The fluorescence intensity was recorded with excitation/emission = 465/580 nm during a temperature scan from from 25 °C to 95 °C with a temperature ramping rate of 1 °C/min. All measurements were performed in triplicate. The raw data were exported to MS Excel and analysis was performed using a custom script provided by Structural Genomics Consortium, University of Oxford. The temperature ranges over which protein unfolding occurred was established at temperatures below the maximum fluorescence intensity. The processed data were fitted to the Boltzmann equation by linear regression analysis.

***Molar Extinction Coefficient and in vitro SPF Calculations***

The photoprotection of palythine was assessed by various *in vitro* methods based on the spectral profile of palythine between 290-400nm. The first test is the calculation of the molar absorption coefficient (ε^(m)^) at λ_max_. *In vitro* protection factors of palythine were measured by BASF GmbH using the methods described by Herzog and Osterwalder ^2^. Briefly, this used the extinction coefficient at various wavelengths across the UVR spectrum (280-400nm) to calculate the SPF and UVA-PF (Table S1). Palythine provided good SPF values but poor UVA-PF values, in line with its spectral properties and high ε^(m)^.

**References**

1 Niesen FH, Berglund H, Vedadi M. The use of differential scanning fluorimetry to detect ligand interactions that promote protein stability. *Nature protocols* 2007; **2**: 2212-21.

2 Herzog B, Osterwalder U. Simulation of sunscreen performance. In: *Pure and Applied Chemistry*, Vol. 87. 2015; 937.
