# Supplementary material for: Molecular photoprotection of human keratinocytes in vitro by the naturally occurring mycosporine‐like amino acid palythine
Source: Br J Dermatol. 2018 Mar 25;178(6):1353–63. doi: 10.1111/bjd.16125 (PMC6032870; doi:10.1111/bjd.16125)
Supplement: Supplementary file 2 — Table S1. The molar extinction coefficient and in vitro sun protection factor of palythine. [file BJD-178-1353-s002.docx]

**Table S1 The molar extinction coefficient and *in vitro* sun protection factor of palythine.** The average molar extinction coefficient of palythine ± SD is displayed, calculated from n= 3. The *in vitro* SPF and UVAPF of palythine was calculated as described in the methods from the absorption spectrum of palythine at a range of concentrations.

| **Endpoint** | **Palythine Conc. (% w/v)** | **SPF** | **UVAPF** |
| --- | --- | --- | --- |
| **In Vitro SPF** | **0.3** | **1.9** | **1.1** |
|  | **1** | **3.4** | **1.2** |
|  | **5** | **10.9** | **1.4** |
|  | **10** | **17.9** | **1.6** |
|  | **25** | **30.2** | **2.1** |
| **Molar Extinction Coefficient (L mol^-1^ cm^-1^)** | **36,947.7 ± 2238.6** | | |
